# The Calcium Channel α2δ1 Subunit: Interactional Targets in Primary Sensory Neurons and Role in Neuropathic Pain

**DOI:** 10.3389/fncel.2021.699731

**Published:** 2021-09-30

**Authors:** Wenqiang Cui, Hongyun Wu, Xiaowen Yu, Ting Song, Xiangqing Xu, Fei Xu

**Affiliations:** ^1^Department of Neurology, Affiliated Hospital of Shandong University of Traditional Chinese Medicine, Jinan, China; ^2^Department of Geriatric Medicine, Affiliated Hospital of Shandong University of Traditional Chinese Medicine, Jinan, China

**Keywords:** neuropathic pain, primary sensory neuron, Cavα2δ1, molecular target, peripheral sensitization

## Abstract

Neuropathic pain is mainly triggered after nerve injury and associated with plasticity of the nociceptive pathway in primary sensory neurons. Currently, the treatment remains a challenge. In order to identify specific therapeutic targets, it is necessary to clarify the underlying mechanisms of neuropathic pain. It is well established that primary sensory neuron sensitization (peripheral sensitization) is one of the main components of neuropathic pain. Calcium channels act as key mediators in peripheral sensitization. As the target of gabapentin, the calcium channel subunit α2δ1 (Cavα2δ1) is a potential entry point in neuropathic pain research. Numerous studies have demonstrated that the upstream and downstream targets of Cavα2δ1 of the peripheral primary neurons, including thrombospondins, *N*-methyl-D-aspartate receptors, transient receptor potential ankyrin 1 (TRPA1), transient receptor potential vanilloid family 1 (TRPV1), and protein kinase C (PKC), are involved in neuropathic pain. Thus, we reviewed and discussed the role of Cavα2δ1 and the associated signaling axis in neuropathic pain conditions.

## Introduction

Neuropathic pain is a chronic pain triggered by peripheral nerve injury, postherpetic neuralgia, diabetic neuropathy, and chemotherapeutic agents, such as cisplatin (Colleoni and Sacerdote, 2010). Its prominent symptoms are spontaneous pain, hyperalgesia (increased pain sensitivity from a painful stimulus), and allodynia (pain in response to a non-painful stimulus) (Kim et al., 2014). These symptoms lead to prolonged discomfort in patients. It is calculated that 9.8% of the population in the United States experiences neuropathic pain (Yawn et al., 2009). Because the molecular mechanisms of neuropathic pain have not been fully elucidated, the management of neuropathic pain remains challenging.

Currently, the commonly used drugs for neuropathic pain include antiepileptics, antidepressants, anticonvulsants, opioid analgesics, and *N*-methyl-D-aspartate receptor (NMDAR) antagonists (Amato et al., 2019). Gabapentin (GBP), a second-generation antiepileptic drug, is one of the first-choice drugs for the treatment of neuropathic pain (Moore and Gaines, 2019). It has good curative effect on diabetic neuralgia, postherpetic neuralgia, trigeminal neuralgia, and sciatica neuralgia. However, the adverse effects of gabapentin are significant, and its precise mechanism of action is uncertain. Many lines of evidence have demonstrated that voltage-gated Ca^2+^ channels were directly blocked by GBP binding to its α2δ1 subunit (Grice and Mertens, 2008; Skubatz, 2019). That leaded to the reduction of presynaptic Ca^2+^ influx and the decreased synaptic release of glutamate, which is the major excitatory neurotransmitter of the brain (Grice and Mertens, 2008; Skubatz, 2019). Thus, the calcium channel α2δ1 subunit is a potential target for the treatment of neuropathic pain. To develop novel effective anti-neuropathic pain agents with fewer side effects, it is promising to understand the α2δ1-related mechanisms and reveal new mechanism-specific treatment targets.

The calcium channel α2δ subunits were first described as accessory subunits of voltage-gated calcium channels (VGCCs) (Gurnett and Campbell, 1996). Four α2δ subunits have been cloned: α2δ1, α2δ2, α2δ3, and α2δ4. The α2δ1 subunit is the receptor of GBP and certain thrombospondins (TSPs). It is expressed in neurons and their axonal terminals and dendrites throughout the central and peripheral nervous system (CNS and PNS) (Eroglu et al., 2009). The TSPs secreted by astrocytes exert their synaptogenic effects by binding to their neuronal receptor α2δ1 (Wang et al., 2020). TSP–α2δ1 interactions participate in synaptic development and neuropathic pain (Risher et al., 2018). Due to its very short cytoplasmic tail, α2δ1 is not able to active intracellular signaling by itself, while its extracellular domain almost could functionally mimic the full-length α2δ1 during synapse formation (Eroglu et al., 2009). Thus, Cavα2δ1 is linked to intracellular signaling via other membrane proteins and is involved in the occurrence and development of neuropathic pain by interacting with several molecules.

This review will cover several novel findings concerning the calcium channel α2δ1 subunit and α2δ1-related proteins of the PNS, particularly their roles in neuropathic pain. This review will aid in the better understanding of the peripheral mechanisms of neuropathic pain and develop novel effective anti-neuropathic pain agents with fewer side effects.

## The α2δ1 Subunit and Its Role in Neuropathic Pain

In the human genome, four α2δ proteins (α2δ1–α2δ4) are encoded by four genes (*CACNA2D1*–*CACNA2D4*), respectively (Dolphin, 2012). Four different α2δ isoforms have selective effects on the level of functional expression and the voltage dependence of different α1 subunits (Davies et al., 2007). Among the genes encoded for α2δ, the *CACNA2D1* may be appropriate for drug development to alleviate neuropathic pain. The upregulated expression of *CACNA2D1* contributed to chronically maintain neuropathic pain by enhancing VGCCs activation, neuronal excitability, and neurotransmission (Yusaf et al., 2001; Gribkoff, 2006; Li et al., 2006, 2014). Moreover, *CACNA2D1* could modulate CGRP and AC-PKA/protein kinase C (PKC)/MAPK signaling pathways in the dorsal root ganglion (DRG) in chronic pain process (Sun et al., 2020). Fundamentally, *CACNA2D1* gene performs these functions by the genetic translation product—the α2δ1 protein. In addition, the expression of α2δ1 is also regulated by epigenetic factors. The histone deacetylase inhibitors—JNJ-26481585—significantly upregulated the expression of α2δ1 subunit in spinal cord and induced mechanical hypersensitivity in mice (Capasso et al., 2015). MicroRNA-183 cluster (miR-183/96/182) controlled more than 80% of neuropathic pain-regulated genes and scaled mechanical pain sensitivity in mice by regulating the expression of α2δ1 and α2δ2 (Peng et al., 2017). Meanwhile, human *CACNA2D1* and *CACNA2D2* genes of DRG are predictive targets of miR-183 cluster. Bioinformatic analysis revealed that miR-107 had a potential binding site for the α2δ1 encoding gene *CACNA2D1*. In laryngeal cancer cells, miR-107 decreased the expression levels of *CACNA2D1* (Huang C. et al., 2020). Thus, epigenetic mechanisms, such as histone posttranslational modification and microRNA, may play an important role in mediating the expression of α2δ1 and chronic neuropathic pain, as they are instructive for transcription, and potentially long lasting.

**FIGURE 1:**
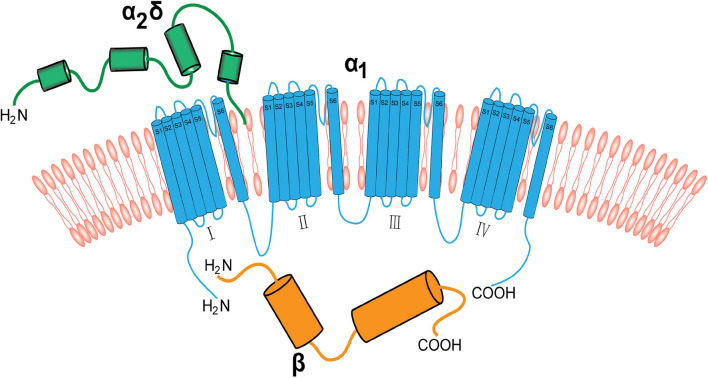
The neuronal voltage-gated calcium channel complex. Voltage-gated calcium channels include the transmembrane pore-forming α1 subunit (red), extracellular α2δ (green), and cytoplasmic β (blue) subunits. The α1 subunit is a transmembrane protein linking the α2δ subunit and β subunit. The α2δ subunit is mainly extracellular and binds to the membrane via GPI anchors. The β subunit binds to α1 subunit via the intracellular I–II linker.

The α2δ1 subunit is strongly expressed in the cardiac and smooth muscles, and is likely to be the main α2δ subunit associated with calcium channels in these tissues (Bikbaev et al., 2020). In addition, the α2δ1, a structural subunit of the VGCCs, is strongly expressed in the nervous system. The core complex of neuronal VGCCs consists of an α1 subunit, which contains the ion-conducting pore, and the auxiliary β and α2δ subunits. The α1 subunit is a transmembrane protein linking α2δ and β. It consists of four transmembrane regions (I–IV) that have the same transmembrane structure. Each transmembrane structure has six transmembrane helices (S1–S6). The vast majority of the α2δ segment is extracellular and most likely linked to the plasma membrane via glycosyl-phosphatidylinositol (GPI) anchors. The β subunit is the intracellular component and binds the intracellular I-II linker of α1 subunits at the so-called α-interaction domain (Figure 1; De Waard et al., 1995; Davies et al., 2010; Catterall, 2011; Geisler et al., 2015).

In the CNS, α2δ1 is present in neurons, axonal terminals (Marais et al., 2001), and dendrites (Eroglu et al., 2009). α2δ1 is correlated with excitatory rather than inhibitory neurons (Bikbaev et al., 2020). It is mainly present in presynaptic terminals and, to a much lower extent, in the cell bodies of neurons in many brain regions under physiological conditions (Alles and Smith, 2018; Bikbaev et al., 2020). In the PNS, α2δ1 is strongly expressed in all neuronal cell types of DRG and trigeminal ganglion (TG), such as IB4^+^, CGRP^+^, and NF200^+^ neurons (Tachiya et al., 2018; Cui et al., 2020). It was found that α2δ1 was expressed in most neurons of different sizes after nerve injuries, predominantly in small (less 20 μm) and medium (20–50 μm) neurons in animal models of sciatica neuralgia and trigeminal neuralgia (Tachiya et al., 2018; Cui et al., 2020). In addition, studies using rat models of chronic constriction injury to the infraorbital nerve (CCI-ION) have shown that the number of α2δ1-immunoreactive neurons over 25 μm in diameter was obviously increased (Li et al., 2014). The α2δ1 subunit was increased in the neurons of the DRG and TG and was mainly associated with terminal fields, rather than cell bodies, after nerve injury (Bauer et al., 2010). Research has shown that α2δ1 was synthesized in the neuron cell bodies and then trafficked to the plasma membrane of the DRG presynaptic terminals that terminate in the spinal cord dorsal horn (Bauer et al., 2009). Meanwhile, the ipsilateral myelinated and non-myelinated DRG axons were increased after partial sciatic nerve ligation and the α2δ1 accumulated at the ligation site (Bennett et al., 2003). In a rat model of CCI-ION, the α2δ1 and Vglut_2_-positive (Vglut_2_^+^) puncta (excitatory presynaptic glutamatergic terminal) were increased in the superficial dorsal horn of the cervical spinal cord, and all increased terminals were α2δ1^+^ (Li et al., 2014). These studies indicated that α2δ1, which was trafficked and accumulated in the superficial dorsal horn of the spinal cord, regulated the excitatory synaptogenesis. However, the mechanisms of trafficking and accumulation of α2δ1 in neuropathic pain remain unclear. If the key target is found to block α2δ1’s transmission and accumulation at the terminal and prevent the generation of abnormal excitatory synapses, the occurrence and development of neuropathic pain will be attenuated.

Several studies have demonstrated that the expression level of α2δ1 was elevated in the TG after partial transection of the infraorbital nerve (pT-ION) (Cui et al., 2020) and CCI-ION (Li et al., 2014). The mechanical and cold hyperalgesia induced by nerve injury could be mimicked by injecting the Cavα2δ1 overexpression adeno-associated virus (AAV) and be reversed by injecting the AAV of Cavα2δ1 downregulation in TG neurons (Cui et al., 2020). These results indicated that the Cavα2δ1 of primary afferent neurons play a vital role in neuropathic pain and that is mainly related to its regulation of Ca^2+^ inflow.

However, the regulation of Ca^2+^ influx of α2δ1 is different from that of the well-studied TRP channels and NMDA receptors in neuropathic pain. TRPs and NMDA receptors can directly mediate the Ca^2+^ influx when they are activated, but α2δ1 indirectly. α2δ1, an accessory subunit of VGCCs, is fully exposed to the extracellular environment and anchored outside the plasma membrane by a phosphatidylinositol. For one thing, nociceptive stimuli induced the upregulation of the expression of α2δ subunit. That regulated the transport, localization, and biophysical properties of α1 subunit and induced the fast assembly of VGCCs (Gurnett et al., 1997; Brodbeck et al., 2002). These processes triggered the influx of Ca^2+^ and the increased excitability of neurons. For another, α2δ1 can bind to other calcium channels through the binding site and then induce the Ca^2+^ inflow. Thus, the study of α2δ1-related targets is important to understand the role of α2δ1 in neuropathic pain.

## The α2δ1 Subunit Is the Neuronal Thrombospondins Receptor Responsible for Synaptogenesis

Thrombospondins are expressed in various cell types and play various roles in cell signaling; these consist of extracellular, oligomeric, multi-domain, and calcium-binding glycoproteins (Adams and Lawler, 2011). They are present in various tissues and consist of five members, TSP 1–5 (Sipes et al., 2018). For example, platelets, endothelial cells, skeletal muscles, fibroblasts, neurons, and astrocytes each express one or more TSP isoforms (Christopherson et al., 2005; Sipes et al., 2018). In mammals, TSPs have many functions, such as wound healing, angiogenesis, and synaptogenesis (Adams and Lawler, 2011; Ponticelli and Anders, 2017).

**FIGURE 2:**
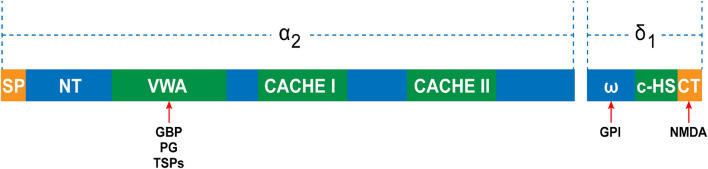
The domain structure of the α2δ1 subunit. The α2 peptide contains N-terminal domain, VWA domain, and two extracellular domains (CACHE I and II). The VWA domain is the potential binding site for gabapentin (GBP), pregabalin (PG), and thrombospondins (TSPs). The δ peptide contains ω amino acids, to which the glycosyl-phosphatidylinositol (GPI) anchor can attach, C-terminal hydrophobic sequence (c-HS), and C-terminal, which is the NMDA binding site.

Studies have demonstrated that the expression levels of TSPs were thoroughly altered in pathophysiological conditions (Chen et al., 2000; Adams and Lawler, 2011). Particularly, the expression of TSP4 was increased in the DRG after peripheral nerve injury (Pan et al., 2015; Risher et al., 2018). Thus, TSP4 of PNS is the protein of focus in neuropathic pain research. Risher et al. showed that elevated targeting of α2δ1 by TSP4 contributed to hypersensitivity of the peripheral sensory systems by decreasing the activation of high-voltage calcium currents while increasing low voltage-activated calcium currents in DRG neurons (Risher et al., 2018). TSP4 promotes the assembly of excitatory glutamatergic synapses by binding to α2δ1 in the CNS (Crosby et al., 2015; El-Awaad et al., 2019). GBP antagonizes the binding of TSPs to α2δ1 and powerfully inhibits excitatory synapse formation *in vitro* and *in vivo* (Eroglu et al., 2009; Lana et al., 2016). Once α2δ1 is activated by TSP, the inter- and intracellular signaling events subsequently occur, which trigger the gathering of synaptic adhesion and scaffolding molecules at nascent synaptic sites. Overexpression of α2δ1 in the absence of TSPs enhances calcium channel surface expression, but does not lead to any changes in the number of synapses (Eroglu et al., 2009). Overexpression of TSPs increases synapse formation but is blocked in α2δ1 knockout mice (Eroglu et al., 2009). There is an interaction between the VWF-A domain of α2δ1 and the epidermal growth factor of all TSPs (Figure 2; Eroglu et al., 2009). Drugs directed toward the VWF-A domain of α2δ1 exert the synaptogenic function of TSPs (Eroglu et al., 2009). This indicates that the binding of TSPs and α2δ1 is the key to synapse formation. Kim et al. (2012) showed that nerve ligation induces upregulation of TSP4 in spinal astrocytes and the enhancement of excitatory synaptic transmission in the dorsal horn, whereas spinal TSP1 and TSP2 exhibit no changes following injury. According to this study, we can assume that increased α2δ1 subunits in the peripheral ganglion transfer from cell bodies to the central terminal and accumulate at the central terminal spinal region when under the influence of elevated TSP4 levels after nerve injury. This progress would promote synaptogenesis between neurons themselves and between neurons and glial cells. Although it has been reported that α2δ1 interacts with TSP to trigger synaptogenesis (Eroglu et al., 2009), a study demonstrated that α2δ1 and thrombospondin had a weak interaction at the membrane surface (Lana et al., 2016). Thus, more work remains to be carried out to improve our knowledge of the role of TSP4-α2δ1 signaling in synaptogenesis in neuropathic pain, particularly in the PNS.

## The α2δ1-*N*-Methyl-D-Aspartate Receptor Complex Is Essential for the Activation of Presynaptic *N*-Methyl-D-Aspartate Receptor in Neuropathic Pain

Although NMDARs are conventionally expressed in postsynaptic neurons (Zhou et al., 2011), they are also expressed presynaptically, particularly in primary sensory neurons of the TG and DRG and in their central terminals in the spinal dorsal horn (SDH) (Li et al., 2016; Xie et al., 2016). Pre- and postsynaptic NMDARs play an indispensable role in many physiological and pathological processes, such as the release of neurotransmitters, synaptic plasticity, and neuropathic pain (Li et al., 2016; Xie et al., 2016; Deng et al., 2019; Huang Y. et al., 2020). The reason for this is that NMDARs are essential for the coordinated activity of pre- and postsynaptic neurons (Mayer et al., 1984). In the physiological state, the NMDARs of the TG and DRG are functionally inactive and do not facilitate the release of neurotransmitters (Yan et al., 2013; Deng et al., 2019). However, NMDARs of the central terminals of primary sensory neurons become tonically active in chronic neuropathic pain conditions (Yan et al., 2013). Activation of presynaptic NMDARs induces calcium influx and vesicle exocytosis, causing neuronal depolarization and neurotransmitter release into the SDH (in particular, glutamate) (Yan et al., 2013; Vandresen-Filho et al., 2015). Increased glutamate release from primary afferent terminals to SDH neurons is essential for the synaptic plasticity in neuropathic pain induced by nerve injury and chemotherapy (Yan et al., 2013). Meanwhile, the activation of NMDARs induces activity-dependent DNA double-strand breaks and the following transcription of several neuronal immediate-early genes (Madabhushi et al., 2015; Hagenston et al., 2020). That is important for synaptic plasticity and sensitization of neurons in chronically maintained neuropathic pain.

The activity of NMDARs is regulated mainly by their own phosphorylation status and/or the phosphorylation status of their interacting targets. The α2δ1 subunit directly interacts with NMDARs and forms a heteromeric complex through its C-terminal domain (Figure 2). This induces the activation of presynaptic NMDARs by facilitating synaptic targeting and trafficking of α2δ1–NMDAR complexes at primary afferent terminals in neuropathic pain caused by nerve injury and chemotherapy (Chen et al., 2018, 2019; Deng et al., 2019). The α2δ1–NMDAR complex in the SDH was significantly increased in rats with spinal nerve ligation (SNL) (Chen et al., 2018). The α2δ1 subunit knockout and intrathecal injection of α2δ1 C termini-interfering peptides or pregabalin reversed the synaptic NMDAR hyperactivity associated with neuropathic pain by interrupting the α2δ1–NMDAR interaction and complex formation (Deng et al., 2019). Thus, increased synaptic expression of the α2δ1–NMDAR complex is essential for the enhancement of the activity of synaptic NMDARs in neuropathic pain.

Chemotherapeutic drugs, such as paclitaxel, are known to potentiate nociceptive input by inducing the tonic activation of presynaptic NMDARs, but not postsynaptic NMDARs in the SDH (Xie et al., 2016; Chen et al., 2019; Deng et al., 2019). Given that α2δ1 is involved in surface trafficking as a highly glycosylated protein, the chemotherapy or nerve injury induced excess of α2δ1 protein at primary afferent terminals may facilitate its interaction with NMDARs and promote synaptic expression of α2δ1–NMDAR complexes (Chen et al., 2018; Deng et al., 2019). Chemotherapy induces the assembly of α2δ1 and causes increases in α2δ1–NMDAR complexes at central terminals, causing persistent increases in glutamatergic nociceptive input to postsynaptic SDH neurons, thereby incurring chronic neuropathic pain (Chen et al., 2019). α2δ1 knockout and the inhibition of α2δ1 trafficking by pregabalin or by disrupting α2δ1–NMDAR interactions with α2δ1 C-termini-interfering peptides completely normalized the paclitaxel-induced NMDAR-dependent glutamate release from primary afferent terminals to postsynaptic SDH neurons and significantly attenuated neuropathic pain (Chen et al., 2019). Overexpression of α2δ1 in neurons results in an increased frequency (but not amplitude) of mEPSCs that were induced by NMDA receptors upon depolarization. In addition, selective blocking of the GluN2A subunits of NMDARs decreases the frequency of mEPSCs evoked from SDH neurons in paclitaxel-induced neuropathic pain rats (Xie et al., 2016). Paclitaxel failed to increase the frequency of mEPSCs by dorsal root stimulation in α2δ1 knockout mice (Chen et al., 2019). These results indicated that a transition from being α2δ1-free to α2δ1-bound NMDAR is essential for the activation of presynaptic NMDARs in the SDH and the increase in glutamate release from the central terminals of presynaptic neurons. Targeting α2δ1-bound NMDARs (not the physiological α2δ1–free NMDARs) may be a new promising strategy for treating neuropathic pain. Unlike VGCCs, the NMDARs is also permeable to Na^+^ and K^+^ except for Ca^2+^ (Zhou et al., 2011). Thus, whether the α2δ1–NMDAR interactions can regulate NMDA-dependent cation inflow is also the focus of neuropathic pain research.

## Transient Receptor Potential Vanilloid Family 1 and Transient Receptor Potential Ankyrin 1 Are Interactional Targets of the α2δ1 Subunit in Primary Sensory Neurons

The transient receptor potential (TRP) channel family was first discovered in Drosophila and proposed as a Ca^2+^ permeable channel/transporter (Bloomquist et al., 1988). This discovery led investigators to focus on the study of Ca^2+^ signaling in TRP channels (Zhu et al., 1996; Minke and Parnas, 2006). The high Ca^2+^ selectivity of TRP channels is due to their crucial amino acid residues (Hardie and Minke, 1992). Thus, TRP channels play an important role in the understanding of sensory nerve function in pain conditions (Silverman et al., 2020; Aleixandre-Carrera et al., 2021). TRP channels contribute to many processes, such as the activation of neurons, the release of neurotransmitters from presynaptic central terminals to postsynaptic SDH neurons, and the release of inflammatory mediators (Hung and Tan, 2018; Silverman et al., 2020). Among all TRP channels, transient receptor potential ankyrin 1 (TRPA1) and transient receptor potential vanilloid family 1 (TRPV1) are generally considered to be expressed in nociception-specific neurons (Patil et al., 2020). Thus, they are the two most frequently reported members of pain research.

TRPA1, a cold-sensing channel, is expressed mostly in small-diameter DRG neurons and activated by temperatures lower than 17°C (Peier et al., 2002; Kobayashi et al., 2005; Chukyo et al., 2018). It is a non-selective channel permeable to Ca^2+^, Na^+^, and K^+^, with a much higher permeability to Ca^2+^ compared to other TRPs (Souza et al., 2020). In human, the methylation of a CpG dinucleotide in the promoter of TRPA1 was negatively correlated with the thermal and mechanical pain sensitivity (Gombert et al., 2017). Some studies reported that pharmacological antagonizing and channel silencing of TRPA1 in the peripheral nerves significantly reduced mechanical allodynia as well as chemical and thermal hyperalgesia in different pain models (Honda et al., 2017; Marone et al., 2018). One study indicated that the increased expression of TRPA1 in the cell bodies of TG neurons contributed to migraines induced by glyceryl trinitrate (Marone et al., 2018). Moreover, the TRPA1 receptor antagonist HC-030031 significantly attenuated mechanical allodynia and cold hyperalgesia in a mouse migraine model (Marone et al., 2018). In our research, the expression of TRPA1 was found to be increased in the TG after trigeminal nerve injury and was inhibited by Cavα2δ1 downregulation in the TG (Cui et al., 2020). Importantly, adenovirus-mediated Cavα2δ1 overexpression in TG neurons induced an increase in the expression of TRPA1 in the TG (Cui et al., 2020). These results indicate that TRPA1 in the peripheral nerves contributes to cold, thermal, and mechanical hyperalgesia in neuropathic pain models and that Cavα2δ1 could potentially be an upstream target of TRPA1. A study suggested that the expression of TRPA1 was increased in models of inflammatory and nerve injuries; TRPA1 knockdown reduced cold hyperalgesia but had little effect on mechanical allodynia and thermal hyperalgesia in these models (Obata et al., 2005). Although these data indicated that TRPA1 was involved in cold sensing, the results from TRPA1 knockout mice were confusing; further experimental studies by the authors also failed to clarify the relationship between TRPA1 and cold-sensitive neurons (Obata et al., 2005). Therefore, the role of TRPA1 in the occurrence and development of cold hyperalgesia remains controversial.

Although TRPA1 and α2δ1 are co-involved in the occurrence and development of neuropathic pain, TRPA1 has different cellular localization and functional characteristics. Glial TRPA1 becomes a new focus of neuropathic pain research. Activated Schwann cells TRPA1 induced the generation of reactive oxygen species (ROS) and 4-HNE involved in alcohol-evoked neuropathic pain (De Logu et al., 2019). It suggests that Schwann cells’ TRPA1 is closely related to oxidative stress in neuropathic pain. TRPA1 is activated not only by external stimuli but also by intracellular calcium released by the endoplasmic reticulum (Zurborg et al., 2007; Cavanaugh et al., 2008). Certainly, TRPA1 is important for neuronal calcium homeostasis in both physiological and pathological states. Interestingly, several lines of evidence support that a compensatory expression of TRPA1 could occur after nerve injury; the expression of TRPA1 was downregulated in L5/DRG and upregulated in L4/DRG (Obata et al., 2005; Katsura et al., 2006; Staaf et al., 2009). However, the mechanism of this expression mode is unclear. Further studies are recommended to illuminate the physiological and pathological actions of TRPA1 in sensory transduction and the interaction between TRPA1 and Cavα2δ1.

**FIGURE 3:**
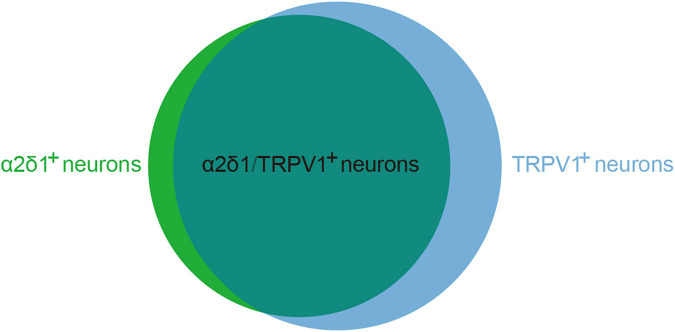
The co-expression proportion of α2δ1 and TRPV1 in primary sensory neurons. α2δ1 and TRPV1 co-expression neurons occupy 85% of α2δ1 immunoreactive neurons (α2δ1^+^) and 64% of neurons that were positive for TRPV1 immunoreactivity (TRPV1^+^).

TRPV1 is predominantly expressed in primary afferent terminals as well as in the cell bodies of DRG and TG neurons. Immunofluorescence results demonstrated that 85% of α2δ1 immunoreactive neurons co-expressed TRPV1, and 64% of neurons that were positive for TRPV1 immunoreactivity also co-expressed α2δ1 (Figure 3; Li et al., 2014; Cakici et al., 2016; Bikbaev et al., 2020). These findings suggest a functional relationship between TRPV1 and α2δ1 in primary sensory neurons. One study demonstrated that gabapentin regulated mosquito allergens induced itching by acting on the voltage-dependent calcium channels’ α2δ1 subunits, which were located in the TRPV1-positive sensory neurons in the peripheral nerve system (Akiyama et al., 2018). Although TRPA1 channels occupy advantageous positions to allow gabapentin to infiltrate primary afferent terminals, it is still unclear whether the activation and opening of TRPV1 channels in peripheral nerve endings effectively transport GBP to its presumed position of action in primary afferent terminals. Future investigations are required to verify this question.

Like VGCCs, TRPV1 activation also mediates Ca^2+^ influx. However, TRPV1 is also subject to calcium-dependent desensitization (Tominaga et al., 1998), which may involve interaction between Ca^2+^-calmodulin and one or more cytoplasmic regions at the N and C termini of the channel (Numazaki et al., 2003; Rosenbaum et al., 2004; Lau et al., 2012). In addition, TRPV1 is mainly expressed in unmyelinated, slowly conducting primary neurons (C-fibers) (Tominaga et al., 1998; Kobayashi et al., 2005). Activation of these fibers promotes the release of proinflammatory factors. Indeed, TRPV1 has come to represent the preeminent molecular marker for defining the nociceptor subpopulation that accounts for sensitivity to thermal stimulation, neurogenic inflammation, and low pH environment (Julius, 2013). Remarkably, 30–50% of TRPV1-expressing neurons also express TRPA1 and TRPV1 interacts with TRPA1 to form a heteromeric channel (Kobayashi et al., 2005; Fischer et al., 2014). In human, neuropathic pain was associated with the specific single-nucleotide polymorphism of TRPV1 and TRPA1 channels (Binder et al., 2011). Rare genetic variants in the genes coding for TRPA1 and TRPV1 had also been found in patients with erythromelalgia (Zhang et al., 2014). Therefore, TRPV1 may be involved in many physiological and pathological processes by interacting with TRPA1. Of course, more research is needed to confirm these.

## Cavα2δ1 Contributes to Neuropathic Pain by Activating Its Downstream Protein Kinase C Target

PKC, the family of serine/threonine kinases, is composed of 11 different isoenzymes and divided into many subtypes (Nishizuka, 1988). This family is calcium-dependent and activated by phosphatidylserine (PS), diacylglycerol (DAG), and protein–protein interactions (Newton, 2010). Different PKC isoforms exist in different tissue and cell locations. For instance, PKCα is expressed at the outer surface of pre- and postsynaptic vessels, while PKCγ is mainly found in the perikaryal cytoplasm and postsynaptic dendrites in adult rats (Hirai, 2018). Interestingly, PKCs are mainly located in the cell bodies and presynaptic central terminals of DRG-nociceptive neurons with small and medium diameters in the SDH (Ase et al., 1988). All PKC subtypes have an identical carboxyterminal that is highly conserved and linked to a divergent amino-terminal regulatory domain by a hinge region (Hirai, 2018; He and Wang, 2019). Normally, PKCs are self-inhibited by a pseudo-substrate sequence that appears at the regulatory domain and occupies the substrate binding site (Hirai, 2018). The self-inhibition of PKCs is disturbed when the regulatory domain is transported to the plasma membrane under the effects of second messengers and allosteric effectors, where its phosphorylation domain is then exposed to the target substrates (He and Wang, 2019).

In cultured DRG neurons separated from adult rats, PKC_δ_, a PKC isoform, was activated by paclitaxel in a dose- and time-dependent manner (He and Wang, 2015). PKC_δ_ inhibitors administered intrathecally reduced the excitability of DRG neurons and relieved neuropathic pain in a paclitaxel-induced peripheral neuropathy mouse model (Chen et al., 2011). In response to inflammatory mediators such as bradykinin and substance P (SP), PKCε from primary sensory neurons was transported from afferent nerve terminals to the plasma membrane of nociceptors in neuropathic pain and inflammatory pain models (Zhang et al., 2007; Penniyaynen et al., 2019). It is verified that PKCε regulates nociception through the modulation of the function of TRPV1 (Chen et al., 2011; Liu et al., 2020). The activation of PKCε can phosphorylate the Ser502 and Ser800 sites of TRPV1, which mediate the increase of capsaicin-induced excitability in afferent neurons (Minke and Parnas, 2006; Chen et al., 2011; Liu et al., 2020). Thus, PKC may have significant implications for neuropathic pain. However, it has not yet been determined which proteins PKC interacts with, or if these proteins are being phosphorylated by PKC.

**FIGURE 4:**
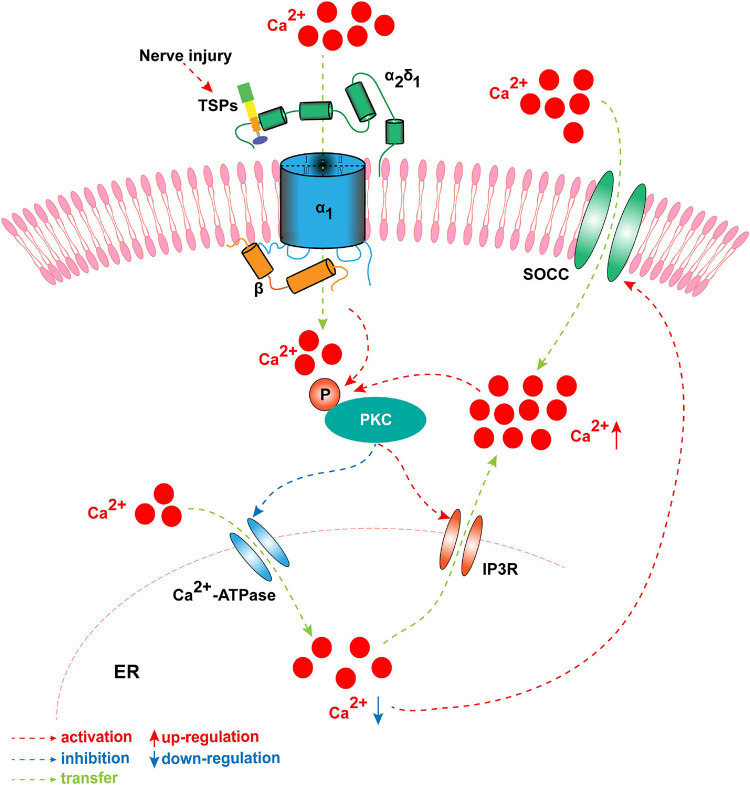
The concentration of intracellular Ca^2+^ is elevated by TSP4 via binding to Cavα2δ1 and activating the downstream PKC signaling pathway when nerve is injured. Once nerve is damaged, TSP4 first binds with the Cavα2δ1 and mediates Ca^2+^ influx, and then induces the phosphorylation of PKC. The p-PKC decreases the function of endoplasmic reticulum (ER) Ca^2+^-ATPase and inhibits Ca^2+^ entering into ER. Meanwhile, p-PKC activates IP3R and allows Ca^2+^ flowing out of ER. It results in the downregulation of ER Ca^2+^ concentration which induces the opening of store-operated calcium channel. These processes successfully cause the influx of Ca^2+^ and the upregulation of intracytoplasmic Ca^2+^. Finally, the intracellular Ca^2+^ further activates primary sensory neurons, which leads to the formation of peripheral sensitization.

TSP4 modulates intracellular Ca^2+^ homeostasis in primary sensory neurons of the TG and DRG by activating PKC signaling and subsequently regulating Ca^2+^ influx and buffering. This process involves the Ca^2+^-ATPase of the sarco-endoplasmic reticulum and plasma membrane after binding to the α2δ1 subunit (Figure 4; Guo et al., 2017). The regulating action of TSP4 on intracellular Ca^2+^ signaling is weakened in conditional knockout Cavα2δ1 mice and is also dependent on the PKC signaling pathway (Guo et al., 2017). These findings indicate that TSP4 contributes to the process of neuropathic pain by increasing the concentration of intracellular Ca^2+^ via binding to Cavα2δ1 and activating the downstream PKC signaling pathway (Figure 4). Cavα2δ1 contributes to the occurrence and development of neuropathic pain by modulating the PKA/PKC/MAPK signaling pathways in the DRG (Sun et al., 2020). GBP significantly attenuates neuropathic pain by binding to Cavα2δ1 and regulating the intracellular PKC signaling pathway in the SDH of rats (Sun et al., 2020). Moreover, our recent study showed that the phosphorylation of PKC was increased in the TG after pT-ION, and the PKC inhibitor GF109203X (0.2 mg/kg) significantly attenuated the mechanical allodynia and cold hyperalgesia induced by trigeminal nerve injury (Cui et al., 2020). The p-PKC elevation induced by nerve injury was inhibited by Cavα2δ1 downregulation in the TG (Cui et al., 2020). Adenovirus-mediated Cavα2δ1 overexpression in TG neurons induced the increased expression of p-PKC in the TG (Cui et al., 2020). Meanwhile, TRPA1 and gap junctions (CX26, CX36, and CX43) are regulated by PKC, the downstream target of Cavα2δ1 (Cui et al., 2020). In the aforementioned study, the amount of Cavα2δ1 bound to either TSP4 or NMDARs increased after pT-ION. The increased binding induces the activation and opening of these channels and then increases the amount of Ca^2+^ flowing into the neurons, and thus triggers a downstream signaling cascade reaction, such as the phosphorylation of PKC. However, the mechanisms of Cavα2δ1/PKC signaling in primary sensory neurons in neuropathic pain remain unclear. The targets of Cavα2δ1/PKC signaling in primary sensory neurons in neuropathic pain have yet to be determined.

## Conclusion and Implication

**FIGURE 5:**
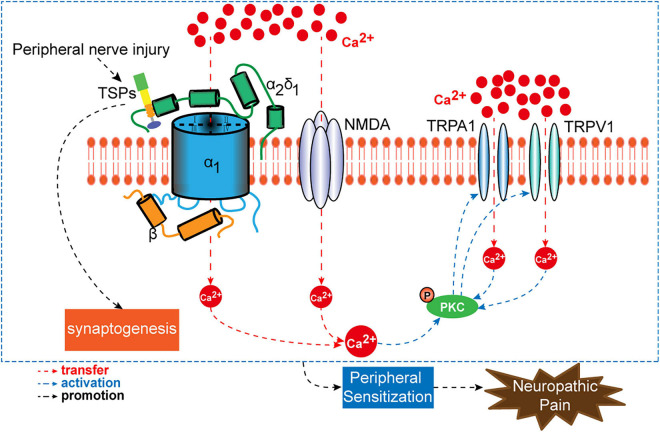
A schematic overview of presynaptic Cavα2δ1 and its interactional targets (TSPs, NMDAR, PKC, TRPA1, and TRPV1) in the process of neuropathic pain.

Neuropathic pain is induced and maintained by the activation of molecular targets that trigger primary sensory neuron sensitization. Current therapies for neuropathic pain are intractable, and these are limited by unclear mechanisms. In clinical practice, many patients with neuropathic pain do not achieve satisfactory pain relief using analgesic drugs. Moreover, the side effects of analgesic drugs are unbearable. Cavα2δ1 is described as an accessory subunit of VGCCs. It contributes to the activation of primary sensory neurons by regulating the entry of Ca^2+^. As discussed above, nerve injury promotes the binding of Cavα2δ1 to either TSP4 or NMDA receptors in primary sensory neurons, which upon activation will increase the influx of Ca^2+^ into the neurons and thus stimulate PKC and the downstream signaling of TRPA1 and TRPV1 channels (Figure 5). These molecules have emerged as alternative targets for the treatment of neuropathic pain. Despite progress being made in terms of understanding the mechanisms of presynaptic Cavα2δ1, our knowledge on its roles and interactions with target sites in the development of neuropathic pain is inadequate. Extensive research is required to further elucidate the genetics and epigenetic mechanisms of Cavα2δ1 in long-lasting neuropathic pain. In addition, the specific intraganglionic administration method requires further validation and subsequent inclusion to clinical practice.

Cavα2δ1 is the important target of gabapentin and pregabalin that are clinically used to treat neuropathic pain (Grice and Mertens, 2008; Chen et al., 2019; Deng et al., 2019; Huang Y. et al., 2020). Recently, mirogabalin, a novel selective ligand for the calcium channel α2δ subunit, is approved for the treatment of neuralgia (Deeks, 2019; Tetsunaga et al., 2020; Zajaczkowska et al., 2021). Drugs targeting α2δ1 for neuralgia are constantly being updated. It will contribute to the development of the more effective drugs to accurately research the mechanisms of α2δ1 and its interactional targets in neuropathic pain via using specific gene interference and genetic methods.

Recent studies demonstrated that calcium channel α2δ1 is a specific candidate marker and therapeutic target for tumor-initiating cells in gastric cancer, hepatocellular carcinoma, and non-small cell lung cancer (Amhimmid et al., 2018; Zhang et al., 2019; Ma et al., 2021). Meanwhile, one study showed that α2δ1 signaling drove cell death, synaptogenesis, circuit reorganization, and gabapentin-mediated neuroprotection in a model of insult-induced cortical malformation (Lau et al., 2017). Therefore, the roles of the calcium channel α2δ1 subunit and its interactional targets are worth investigating in other diseases rather than neuropathic pain.

## Author Contributions

WC and HW wrote the manuscript. XY reviewed the literature. TS modified the language. FX and XX made equal contributions to the drawing of the figures and the revision of the manuscript. All authors contributed to the article and approved the submitted version.

## Conflict of Interest

The authors declare that the research was conducted in the absence of any commercial or financial relationships that could be construed as a potential conflict of interest.

## Publisher’s Note

All claims expressed in this article are solely those of the authors and do not necessarily represent those of their affiliated organizations, or those of the publisher, the editors and the reviewers. Any product that may be evaluated in this article, or claim that may be made by its manufacturer, is not guaranteed or endorsed by the publisher.
